# Hospital Results of a Single Center Database for Stentless Xenograft Use in a Full Root Technique in Over 970 Patients

**DOI:** 10.1038/s41598-019-40772-7

**Published:** 2019-03-13

**Authors:** Jerry Easo, Alexander Weymann, Philipp Hölzl, Michael Horst, Harald Eichstaedt, Ahmed Mashhour, Konstantin Zhigalov, Marcin Szczechowicz, Rohit Philip Thomas, Anton Sabashnikov, Otto E. Dapunt

**Affiliations:** 1 Department of Cardiac Surgery, University Clinic Oldenburg, European Medical School Oldenburg-Groningen, Rahel-Straus Str 10, 26133 Oldenburg, Germany; 2 Department of Cardiac and Thoracic Surgery HELIOS Hospital Siegburg, Ring Str. 49, 53721 Siegburg, Germany; 3 Department of Diagnostic and Interventional Radiology, University Clinic Oldenburg, European Medical School Oldenburg-Groningen, Rahel-Straus Str 10, 26133 Oldenburg, Germany; 4 Department of Cardiothoracic Surgery, Heart Center, University of Cologne, Kerpener Str. 62, 50937 Cologne, Germany; 5 Division of Cardiac Surgery, Medical University of Graz, Auenbrugger Platz 29, 8036 Graz, Austria

## Abstract

Our aim was to analyse the hospital outcome for the worldwide largest series of stentless bioroot xenografts (Medtronic Freestyle) as full root replacement in a single centre over a period of 18 years. Retrospective data analysis was performed for the entire cohort of patients undergoing aortic root surgery with the Medtronic Freestyle valve prosthesis. Logistic regression analysis was performed to analyse predictors of in-hospital mortality. 971 patients underwent aortic full root replacement with the Medtronic Freestyle valve in the period from 1999–2017, with an average age of 68.8 ± 10.3y and gender distribution of 608:363 (male:female). Concomitant surgery was performed in 693 patients (71.4%). In-hospital all-comers mortality was 9.8% (95 patients), with the respective highest risk profiles including dissections (6.4%), endocarditis (5.6%) and re-do procedures (12.5%). In-hospital mortality for elective patients was 7.6% while isolated aortic root replacement demonstrated a mortality of 3.6%. Logistic regression analysis demonstrated age (OR 1.05, p = 0.005), dissection (OR 5.78, p < 0.001) and concomitant bypass surgery (OR 2.68, p < 0.001) as preoperative risk factors for the entire cohort. Postoperative analysis demonstrated myocardial infarction (OR 48.6, p < 0.001) and acute kidney injury (OR 20.2, p < 0.001) to be independent risk factors influencing mortality. This analysis presents a work-through of all patients with stentless bioroot treatment without positive selection in a high-volume clinical center with the largest experience world-wide for this form of complex surgery. Isolated aortic root replacement could be performed at acceptable operative risk for this technically-challenging procedure.

## Introduction

Patients requiring aortic root surgery represent a high-risk cohort due to underlying pathologies, often not amenable to transcatheter valve replacement or rapid deployment valve prostheses. Various conditions such as small aortic annulus, periannular abscess in aortic valve endocarditis or acute dissection involving the aortic root are just a few of clinical scenarios requiring surgical treatment of the anatomical unit of the aortic root, often with inferior results when treated by conventional stented tissue valves^1–4^. Clinical studies have furthermore demonstrated the benefit of root replacement by omission of the obstructive elements of conventional stented valves and facilitated upsizing by at least 1–2 sizes, positively influencing long-term ventricular remodelling and mass regression^5,6^.

At our institution, the Medtronic Freestyle® bioprosthesis is the preferred bioroot valve substitute when performing aortic root surgery^7^. Initial subcoronary implantation technique was rapidly abandoned in favour of the full root procedure due to a majority of patients presenting with root aneurysm or small aortic root with anticipated patient-prosthesis mismatch. This surgical technique could be performed with encouraging initial operative results while increased technical challenge of the root procedure could substantially be reduced by increasing surgical experience for this high-risk patient cohort^7^.

The goal of this clinical report was to perform an in-depth analysis of the surgical experience comparing separate subgroups of over 970 patients, this being the largest database of this stentless xenograft in a full root technique to date, focussing on hospital results and in-hospital mortality. Furthermore, we performed regression analysis to determine factors influencing hospital mortality in order to identify variables to consider when implementing this form of surgery.

## Materials and Methods

This retrospective study analysed 971 patients in a time period of 18 years between November 1999 and March 2017 undergoing aortic root replacement using the Medtronic Freestyle® bioprosthesis. Altogether nine attending surgeons with similar experience in aortic valve and root surgery performed the full root technique.

**Figure 1:**
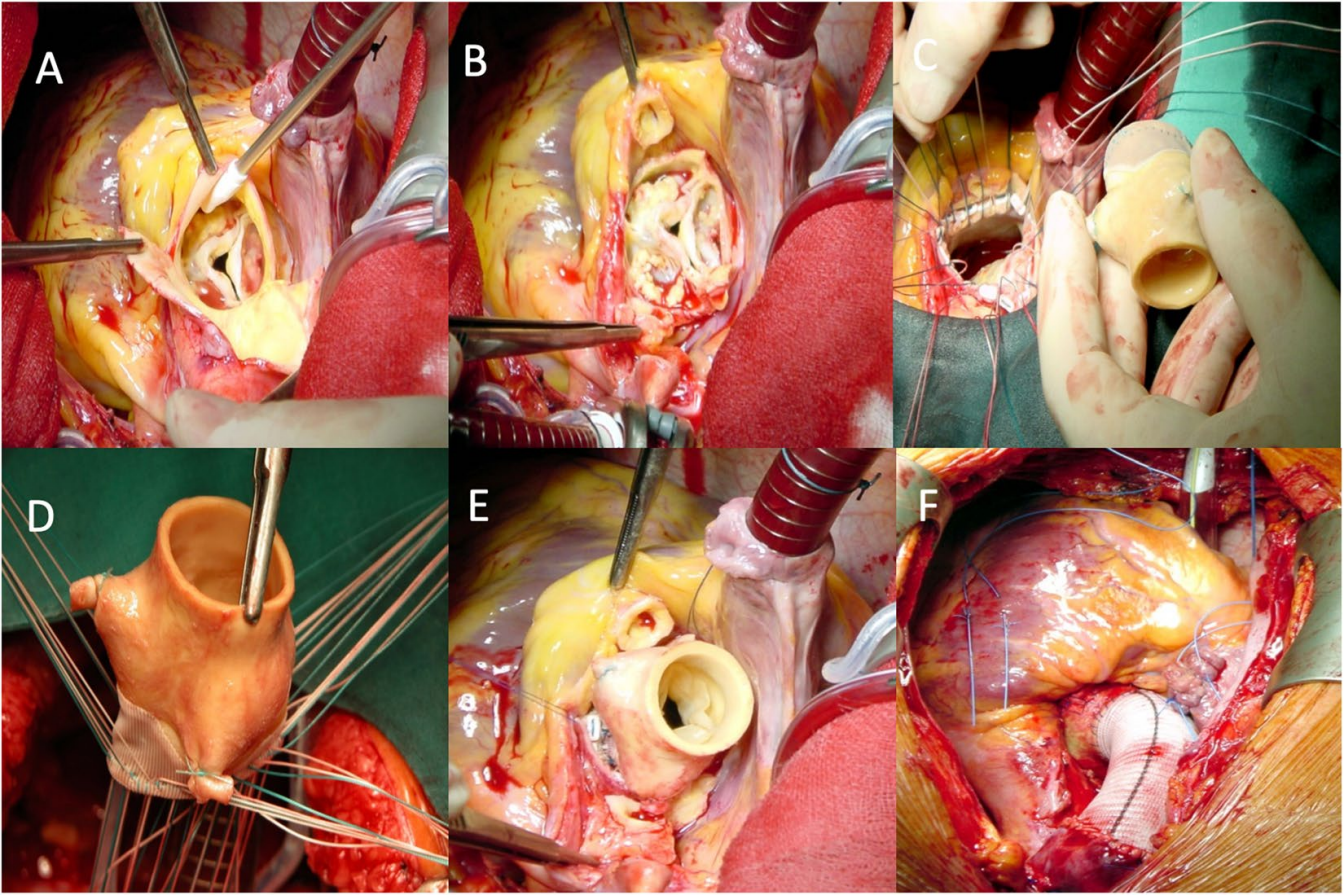
Operative steps for the implantation of the Medtronic Freestyle. (**A**) Resection of the Sinus of Valsalva and application of crystalloid cardioplegia over the coronary ostia. (**B**) Exposure of the heavily calcified aortic valve and isolation of the coronary buttons. (**C**) Placement of pledgetted braided sutures along the aortic annulus for aortic root implantation. (**D**) Passing of braided sutures through the buttressed rim of the stentless valve for aortic root implantation. (**E**) Reimplantation of the coronary ostia into the porcine aortic root using polypropylene sutures after knot-down of the annulus sutures to re-establish myocardial perfusion. (**F**) Replacement of the ascending aorta with a vascular prosthesis in this case of aneurysmatic dilation and placement of epicardial pacemaker wires.

The primary approach was via a median sternotomy with a smaller percentage of patients treated with a minimally-invasive approach using a partial upper sternotomy. Cardiopulmonary bypass was achieved by cannulation of the ascending aorta and 2-stage venous cannulation in cases without involvement of the aortic arch. In patients with Type A aortic dissection, our standard arterial cannulation technique was via the right subclavian artery with use of a vascular prosthetic side branch. Cardioplegic arrest was performed mostly with cold crystalloid cardioplegia and blood cardioplegia in a small group of patients. Implantation of the stentless bioroot was performed with pledgetted sutures for proximal fixation, a smaller group was performed using single sutures to obtain a perfect transition from the left ventricular outflow tract to the bioroot. Coronary button mobilisation was performed and reimplantation was undertaken by a continuous 5–0 polypropylene suture. Rotation of the stentless xenograft to facilitate reimplantation of the coronary buttons was performed in seldom cases. The largest percentage were performed by reimplantation of the left coronary button in the porcine ostium and creation of a neo-ostium for the right coronary ostium. Patients presenting with an aneurysm of the ascending aorta were treated by implantation of a vascular prosthesis (Vascutek®, Renfrewshire, Scotland) interposed between the bioroot and the native ascending aorta. Figure 1 depicts the separate stages of the operative procedure. Concomitant surgery included the whole spectrum such as coronary bypass surgery, closure of atrial and ventricular septal defects, mitral valve surgery, heart rhythm surgery and aortic arch surgery, often in combination with each other. Aortic arch surgery was performed aggressively, with standardized use of moderate hypothermia and antegrade cerebral perfusion when necessary.

Anticoagulation was performed with use of intravenous heparin until removal of chest tube drains and mobilization. Subsequently, low molecular weight heparin was implemented for thrombosis prophylaxis. The antithrombotic therapy recommended upon transferal to rehabilitation or secondary care hospitals was aspirin 100 mg daily. Administration of Warfarin was only recommended in cases with respective indications.

Echocardiographic examination was performed in a standard manner in the initial postoperative period by day 5, mean pressure gradients was calculated by the modified Bernoulli’s equation, correcting for proximal velocity. Regurgitation was recorded as absent, trivial, moderate or severe based on standard criteria including assessment of jet width, circumference and eccentricity.

Statistical analysis of the recorded data was performed using IBM SPSS version 24 for Mac (IBM Corp.). Categorical variables were reported as absolute and percentage values. Continuous variables were showed as mean values ± standard deviation (SD) in cases of normal distribution, or as median values (Mdn) with interquartile range (IQR) in cases of non-normal distribution. We used the univariate logistic regression analysis to assess the predictors of in-hospital mortality. The odds ratio (OR) and 95% confidence intervals (CI) were calculated in relation to each analysed variable. We used the chi-square test to investigate the differences of categorical variables distributions in analysed subgroups.

Clinical data was collected throughout the entire postoperative period, with reporting of adverse events according to the recommended guidelines of the Society of Thoracic Surgery and the American Association of Thoracic Surgery^8^. Hospital mortality was defined as mortality within the initial 30 postoperative days or within the hospital stay when exceeding the first 30 days. The study protocol was approved by the Ethics Committee of the Carl von Ossietzky University Oldenburg (2017–040). The requirement for informed consent from individual patients was waived due to the retrospective nature of the study design. All methods were performed in accordance to the relevant guidelines and regulations.

## Results

From November 1999 to March 2017, 971 patients underwent aortic root surgery with use of the Medtronic Freestyle aortic valve prosthesis. The average age was 68.8 ± 10.3 years with a gender distribution of 363 women and 608 men (37.3:62.7%). The predominant pathology accompanying valve disease was aneurysm of the ascending aorta, demonstrated in 478 patients of 971 (49.2%). Emergent surgery was performed in 119 patients (12.3%). Aortic dissection was the prevalent pathology in 62 patients and infective endocarditis in 54 patients. The remaining 3 patients underwent emergent surgery due to acute  decompensated heart failure, including one bioprosthetic degeneration after 8 years. The further preoperative patient characteristics are listed in Table 1.

**Table 1:** Preoperative patient characteristics.

Characteristics	Whole cohort eARR	ARR	ARR+	p-value
**Demographic data**				
Number of patients	971	197	774	
Age [years]	68.8 ± 10.3	65.7 ± 12.6	69.6 ± 9.5	<0.001
Female	363 (37.4%)	90 (45.7%)	273 (35.3%)	0.007
Body surface area [m^2^]	1.96 ± 0.21	1.96 ± 0.22	1.96 ± 0.21	0.655
**Aortic valve disease**				
Only stenosis	175 (18%)	28 (14.2%)	147 (19%)	0.12
Only regurgitation	321 (33%)	51 (25.9%)	270 (34.9%)	0.017
Stenosis and regurgitation	475 (48.9%)	118 (59.9%)	357 (46.1%)	<0.001
Endocarditis	54 (5.6%)	0	54 (7%)	<0.001
**Aortic disease**				
Aneurysm of the aorta ascendens	478 (49.2%)	96 (48.7%)	382 (49.4%)	0.876
Aortic dissection	62 (6.4%)	0	62 (8%)	<0.001
**Mitral valve disease (> or** = **2. Degree)**				
Only stenosis	2 (0.2%)	0	2 (0.3%)	0.999
Only regurgitation	85 (8.8%)	0	85 (11%)	<0.001
Stenosis and regurgitation	4 (0.4%)	0	4 (0.5%)	0.59
Paravalvular leak	1 (0.1%)	0	1 (0.1%)	0.999
**Comorbidities**				
Coronary artery disease	345 (35.3%)	41 (20.8%)	304 (39.3%)	<0.001
Arterial hypertension	505 (52%)	84 (42.6%)	421 (54.4%)	0.003
Diabetes	63 (6.5%)	10 (5.1%)	53 (6.8%)	0.367
Chronic kidney disease	101 (10.4%)	16 (8.1%)	85 (11%)	0.24
Atrial fibrillation	123 (12.7%)	19 (9.6%)	104 (13.4%)	0.153
Emergency surgical indication	119 (12.3%)	0	119 (15.4%)	<0.001

For further comparative analysis, we allocated three patient subgroups: the entire cohort (eARR) as a reference group, elective isolated aortic root replacement as a stand-alone primary procedure (ARR) and the complementary subgroup of root replacements including reoperations and concomitant surgery (ARR+).

Table 1 demonstrates the ARR group to be younger than the eARR group and to have a higher ratio of female patients (p < 0.05). Furthermore, the ARR group shows a higher percentage of patients with mixed lesions of stenosis/regurgitation in comparison to the eARR group and an understandably lower percentage of CAD (p < 0.001), consisting of insignificant coronary lesions.

Table 2 demonstrates intraoperative data. The average valve size implanted was 25.1 ± 2.3 mm with the valve size distribution of 21 mm (n = 44), 23 mm (n = 192), 25 mm (n = 292), 27 mm (n = 296) and 29 mm (n = 146). A re-do procedure was performed in 121 patients (12.5%). Concomitant surgery was necessary in 693 patients (71.4%), predominantly consisting of replacement of the ascending aorta in 313 patients (32.2%) and bypass surgery in 265 patients (27.3%), among others.

**Table 2:** Intraoperative Data.

Characteristics	Whole cohort eARR n = 971	ARR n = 197	ARR + n = 774	p-value
**Durations [min]**				
Operation Median with 1^st^ and 3^rd^ quartile	202 (168;266)	170 (150;198)	214 (176;290)	<0.001
Cardiopulmonary bypass Median with 1^st^ and 3^rd^ quartile	123 (97;165)	105 (90;128)	129 (101–179)	<0.001
Aortic cross clamp Median with 1^st^ and 3^rd^ quartile	89 (72;110)	80 (68;96)	92 (74–115)	<0.001
**Redo heart surgery**	121 (12.5%)	0	121 (15.6%)	
**Concomitant procedures (also as performed in various combinations)**	693 (71.4%)	0	693 (89.5%)	
CABG	265 (27.3%)	0	265 (34.2%)	
CABG without CAD	43 (4.4%)	0	43 (5.6%)	
Replacement of the ascending aorta	313 (32.2%)	0	313 (40.4%)	
Aortic arch surgery	81 (8.3%)	0	81 (10.5%)	
Mitral valve surgery	297 (30.6%)	0	297 (38.4%)	
Tricuspidal valve surgery	6 (0.6%)	0	6 (0.8%)	
LVOT – myotomy and myectomy	6 (0.6%)	0	6 (0.8%)	
Closure of congenital VSD	1 (0.1%)	0	1 (0.1%)	
Closure of congenital ASD	1 (0.1%)	0	1 (0.1%)	
Pericardectomy	1 (0.1%)	0	1 (0.1%)	
Closure of patent foramen ovale	20 (2%)	0	20 (2.6%)	
**Combination of two or more concomitant procedures**	258 (26.6%)	0	258 (33.3%)	

Table 2, depicting intraoperative data, shows ARR as a stand-alone procedure performed in 197 patients (mean age 69 ± 12.6y, male: female 107:90) with a median cross-clamp time of 80 min (IQ range 68–96) and cardiopulmonary bypass (CPB) time of 105 min (IQ range 90–128). When analyzing the eARR group, a median cross-clamp time of 89 min (IQ range 72–110) and median cardiopulmonary bypass being 123 min (IQ range 97–165) was demonstrated. ARR+ demonstrated a median cross-clamp time of 92 min (IQ range 74–115) and a median cardiopulmonary bypass being 129 min (IQ range 101–179), demonstrating a significant reduction of operative time for isolated ARR patients.

Postoperative adverse outcomes are demonstrated in Table 3. For eARR, permanent neurological impairment was detected in 20 patients (2.1%). Acute kidney injury, defined as patients requiring new onset-dialysis, was present in 66 patients (6.8%). Revision for bleeding was performed in 80 patients, 8.2% of the entire cohort. Postoperative pacemaker surgery was necessary in 2.7% of all operated bioroots. The hospital mortality of all patients was 95/971, a rate of 9.8%. None of these patients died in the operative theater, casualties were mainly within the initial 30-day period with 21 patients within the first postoperative day (including 10 patients with acute dissection and three re-operative procedures). Seven patients died after the initial 30-day period within their hospital stay. Elective operations of the entire cohort presented a mortality rate of 65/852 patients (7.6%). Emergent operations showed a mortality rate of 30/119 patients (25.2%).

**Table 3:** Early outcome and postoperative complications.

Characteristics	Whole cohort eARR n = 971	ARR n = 197	ARR + n = 774	p-value
Chest tube drainage within the first 24 hours [ml] Median with 1^st^ and 3^rd^ quartile	930 (550;1950)	650 (400;1050)	1078 (600;2250)	<0.001
Mechanical ventilation [h] Median with 1^st^ and 3^rd^ quartile	10 (2;18)	0 (2;15)	10(2;20)	0.003
Intensive care unit length of stay [days] Median with 1^st^ and 3^rd^ quartile	2 (1;5)	1 (1;3)	2 (1;6)	<0.001
Hospital length of stay [days] Median with 1^st^ and 3^rd^ quartile	8 (6;12)	7 (5;9)	8 (6;13)	<0.001
Transfusions of packed red cells [units] Median with 1^st^ and 3^rd^ quartile	2 (0;6)	0 (0;3)	2,5 (1;7)	<0.001
**Postoperative adverse events**				
Stroke	20 (2.1%)	0	20 (2.6%)	0.02
Revision for bleeding	80 (8.2%)	7 (3.6%)	73 (9.4%)	0.007
ECMO implantation	24 (2.5%)	2 (1%)	22 (2.8%)	0.198
IABP implantation	38 (3.9%)	2 (1%)	36 (4.7%)	0.014
Pericardial tamponade	31 (3.2%)	3 (1.5%)	28 (3.6%)	0.174
Acute kidney injury requiring dialysis	66 (6.8%)	5 (2.5%)	61 (7.9%)	0.008
Reintubation	35 (3.6%)	4 (2%)	31 (4%)	0.282
Tracheotomy	57 (5.9%)	4 (2%)	53 (6.8%)	0.01
Pacemaker implantation	26 (2.7%)	2 (1%)	24 (3.1%)	0.138
Atrial fibrillation	254 (26.2%)	54 (27%)	200 (25.8%)	0.651
Myocardial infarction	6 (0.6%)	2 (1%)	4 (0.5%)	0.352
CPR	34 (3.5%)	6 (3%)	28 (3.6%)	0.83

ARR as an elective stand-alone root procedure could be performed with an acceptable operative risk with a hospital mortality of 3.6% (7/197 patients). The chest tube drainage loss median was 650 ml (IQ range 400–1050 ml). No patient suffered from neurological impairment, seven patients required re-exploration for bleeding (3.6%), five patients required temporary dialysis for renal failure (2.5%), two patients required postoperative pacemaker surgery (1%) and two patients suffered from postoperative low output syndrome (1%).

The ARR+ group demonstrated similar results to the entire cohort, with significant differences to ARR with respect to almost all important postoperative variables due to the higher risk of this patient cohort.

Subgroup analysis demonstrated a mortality rate of 14.8% (8/54 patients) for patients presenting with acute valve endocarditis. Chi square test demonstrated the form of surgery to be a significant risk for mortality with emergent vs. elective showing p < 0.001, redo surgery calculated likewise to be a significant risk (p = 0.019).

Predictors of in-hospital mortality are demonstrated in Table 4. The eARR vs. ARR vs. ARR+ groups demonstrated age (OR 1.05), aortic dissection (OR 5.78), CABG (OR 2.68) and bailout bypass surgery (OR 7.13) to be significant predictors of mortality. Furthermore, length of operative time correlates to the higher risk of mortality. Moreover, all significant variables such as postoperative renal failure (OR 20.2), rethoracotomy (OR 6.17), myocardial infarction (OR 48.6), postoperative LOS (OR 28.6) are shown, among others, to be significant risk factors for postoperative mortality.

**Table 4:** Predictors of in-hospital mortality, logistic regression.

Characteristics	Whole cohort eARR			ARR			ARR+		
	OR	95%CI	p-value	OR	95%CI	p-value	OR	95%CI	p-value
**Preoperative**									
**Age [years]**	**1.05**	**1.02**–**1.07**	**0.001**			**0.114**	**1.037**	**1.01**–**1.07**	**0.009**
**Male gender**	**0.55**	**0.36**–**0.84**	**0.006**			**0.184**	**0.53**	**0.34**–**0.83**	**0.005**
**Aortic dissection**	**5.78**	**3.25**–**10.29**	<**0.001**		—		**4.93**	**2.72**–**8.83**	<**0.001**
Endocarditis			0.2		—				0.41
**CABG**	**2.68**	**1.74**–**4.14**	<**0.001**		—		**2.22**	**1.42**–**3.47**	<**0.001**
**CABG – number of grafts**	**1.98**	**1.59**–**2.46**	<**0.001**		—		**1.85**	**1.48**–**2.31**	<**0.001**
**CABG without CAD**	**7.13**	**3.7**–**13.7**	<**0.001**		—		**6.08**	**3.15**–**11.7**	<**0.001**
Impaired EF (<50%)			0.57			0.226			0.623
Arterial hypertension			0.44			0.44			0.797
Chronic kidney disease			0.15			0.99			0.119
Atrial fibrillation			0.52			0.68			0.696
Diabetes mellitus			0.22			0.286			0.378
**Intraoperative**									
**Length of the operation [min]**	**1.012**	**1.01**–**1.014**	<**0.001**	**1.014**	**1.002**–**1.026**	**0.026**	**1–012**	**1.01–1.014**	<**0.001**
**Aortic cross clamp [min]**	**1.019**	**1.013**–**1.024**	<**0.001**	**1.027**	**1.004**–**1.05**	**0.019**	**1.017**	**1.01–1.022**	<**0.001**
**Concomitant procedures**	**1.95**	**1.13**–**3.36**	**0.016**	—	—	—			**0.77**
**Postoperative**									
**Acute kidney injury**	**20.2**	**11.6**–**35.2**	<**0.001**	**252**	**21**–**2980**	<**0.001**	**15.4**	**8.6**–**27**	<**0.001**
**Revision due to bleeding**	**6.17**	**3.65**–**10.43**	<**0.001**			**0.159**	**5.7**	**3.3**–**9.8**	<**0.001**
**Myocardial infarction**	**48.6**	**5.62**–**420.66**	<**0.001**			**0.99**	**24**	**2.5–235**	**0.006**
Stroke			0.13			—			0.227
**IABP implantation**	**16.2**	**8.15**–**32.2**	<**0.001**			**0.99**	**12.3**	**6.1**–**24.9**	<**0.001**
**ECMO implantation**	**43.55**	**15.8**–**119.9**	<**0.001**			**0.99**	**32.6**	**11.7–91**	<**0.001**
**Low output**	**28.6**	**13.86**–**58.93**	<**0.001**				**23.6**	**11.3–49**	<**0.001**
**Pericardial tamponade**	**3.4**	**1.48**–**7.85**	**0.004**			**0.99**	**3.3**	**1.4**–**7.8**	**0.006**
Pleural effusion			0.917			0.99			0.946
Reintubation			0.74			0.99			0.784
**Tracheotomy**	**2.36**	**1.18**–**4.73**	**0.015**			**0.056**			**0.079**
**Respiratory failure**	**3.56**	**2.18**–**5.82**	<**0.001**	**15.08**	**2.93**–**77.74**	<**0.001**	**2.9**	**1.7**–**4.8**	<**0.001**
**Cardiopulmonary resuscitation**	**9.65**	**4.74**–**19–67**	<**0.001**	**46.8**	**7.1–306**	<**0.001**	**7.75**	**3.55**–**16.9**	<**0.001**
**Pacemaker implantation**	**4.38**	**1.85**–**10.37**	**0.001**	**31.5**	**1.75–565**	**0.019**	**3.4**	**1.37**–**8.45**	**0.008**
**Packed red cells transfusions [units]**	**1.13**	**1.1**–**1.16**	<**0.001**	**1.18**	**1.06**–**1.32**	**0.003**	**1.12**	**1.09**–**1.15**	<**0.001**
**Length of the mechanical ventilation [h]**	**1.002**	**1.001**–**1.003**	<**0.001**			**0.46**	**1.002**	**1.001–1.003**	**0.001**

## Discussion

At our institution, the Medtronic Freestyle valve was used as the prosthesis of choice in cases of stentless aortic root replacement used solely in a full root technique since implementation in Oldenburg in 1999. The increasing experience with good operative results bolstered our concept of softening the indication to treatment for valve disease with milder aortic pathologies such as root endocarditis with abscess formation, requiring complex root reconstruction. Patients with expected patient-prosthesis mismatch otherwise treated by root enlargement or small valves were another group profiting from the full root valve, easily treated by moderate oversizing of the root prosthesis^7,9–12^. Furthermore, often patients wish for stentless root treatment was the reason for use of the bioroot. These conditions, among others, helped develop our experience in 971 patients, to date the largest series worldwide of the Medtronic Freestyle valve implantation as a full root replacement.

The goal of this data analysis was to perform a thorough work-through of data observed over an 18-year time period, investigating all-comers with their respective risk profiles representing the real-world scenario without selection bias.

Isolated aortic valve replacement can be performed at a low operative risk by treatment with stented tissue valves, with an in-hospital mortality of 3.3% of 9722 patients treated in Germany 2016^13^. These patients represent standard patient care, optimal treatment and the gold-standard that other treatment options have yet to achieve. These results are comparable to the data analyzed in this report, demonstrating a mortality of 7/197 patients undergoing elective isolated aortic root replacement as a primary stand-alone procedure (3.6%). This comparison of valve replacement against root replacement demonstrates larger experience with root surgery to reduce perioperative risk. This is comparable to other root reports; Lehmann *et al*. showing an early mortality of 5.5% for patients, excluding endocarditis, undergoing isolated aortic valve and root replacement^14^ and Etz *et al*. demonstrating excellent results in their analysis of longevity after aortic root replacement, showing a mortality of 1.3% for low-risk patients, comparable to our primary elective isolated procedure^15^. The latter report, however, was confined to quinquagenarians, an age group limited between 50–60. This may explain the larger mortality in our series, which include all patients.

Concomitant procedures were performed in 71.4% of all patients analyzed in this cohort, the majority of which included replacement of the ascending aorta. These findings are comparable to studies presenting aortic root replacement, including 79.6% in the Toronto Root analysis by Lehmann and colleagues, among others^14^. Further concomitant surgery included coronary bypass grafting in 265 patients, presenting with 27.3%. These patients were diagnosed with CHD, requiring bypass surgery. A small number of patients required bailout bypass surgery in cases of ventricular dysfunction, or patients suffering from aortic dissection involving the coronary ostia. Reimplantation of the coronary ostia was performed in almost all cases using the native porcine left ostium for the left coronary artery and creating a neo-ostium in the stentless prosthesis at the corresponding position for the right coronary artery. Filling of the ventricle and subsequent torque on the passage of the right ostia was sometimes observed and then rapidly treated by bypass grafting to the right coronary artery. We feel obliged to point out the necessity of utmost care in the technical considerations while performing this type of surgery^16^.

The combination of two or more concomitant procedures was performed in 258 patients (26.6%). This further demonstrates the higher-risk cohort of patients investigated in this retrospective analysis.

Revision for bleeding was performed in 80 patients, 8.2% of the entire cohort. This cohort includes all-comers, including aortic dissections, acute endocarditis patients and re-do procedures, possibly explaining the rather high rate of rethoracotomies. Postoperative pacemaker surgery was necessary in 2.7% of all operated bioroots, possibly explained by the aggressive debridement and large rate of reconstructive procedures at the annular level.

The Medtronic Freestyle root was implanted in 54 patients presenting with acute aortic valve endocarditis. Of these 54 patients 28 had a prosthetic valve and 26 presented with native valve endocarditis. This high rate of prosthetic valve endocarditis is comparable to date described by Leontyev *et al*. showing 55.8% of NVE and 44.2% of PVE from their series of 172 patients undergoing surgery for paravalvular root abscess, treated by valve or root replacement in 70 patients^17^. Musci *et al*. demonstrated likewise a high percentage of PVE with 26.5% of their 221 patients undergoing homograft root replacement^18^. The mortality rate of 14.8% demonstrated in this data analysis reflects on the high-risk profile of these patients and is comparable to studies focusing on the treatment of aortic root endocarditis with tissue valve prostheses^17–21^. The main cause of in-hospital mortality is, as expected, sepsis-related multi-organ failure.

Logistic regression analysis demonstrates a number of pre-existing co-morbidities influencing the prediction of in-hospital mortality. Age at surgery and pre-operative pathologies such as aortic dissection (OP 5.78, p < 0.001) prove to be a significant risk. Necessity of CABG in the absence of CHD naturally shows a major relevance influencing early mortality with an OR of 7.13 and a 95% CI of 3.7–13.7. Interestingly, typical risk factors such as chronic renal disease or impaired ejection fraction failed to demonstrate significance in the prediction of mortality. Cardiopulmonary bypass time, aortic cross clamp time (p < 0.001) and concomitant procedures (p < 0.016) are significant risk factors, likewise demonstrated in large clinical series investigating root replacement. Emergent surgery demonstrates a significance when compared to elective cases (p < 0.001).

In conclusion, it must be clearly stated that the results represent patients requiring root surgery and not merely aortic valve replacement. Root enlargement for small aortic roots has demonstrated an increased operative risk even in the hands of the most experienced^22^ and can clearly be treated by this form of surgery by oversizing and supraannular placement. There should be furthermore no attempt to compare the results of this group of patients with TAVR or stented AVR due to the complexity of the underlying pathology of the root and their varying indications for implantation as a root procedure.

The limitations of this study are clear, the retrospective nature of the data analysis may influence bias. Ongoing data acquisition will provide further information concerning long-term performance of the valve prostheses.

## Conclusion

This data analysis demonstrates this technically challenging operation to be performed at an acceptable operative risk for patients suffering under a multitude of complex root, valve and aortic pathologies at a high-volume center. Isolated aortic root replacement as a primary stand-alone procedure is performed with excellent operative and postoperative results, comparable to valve replacement with stented valve prostheses. The integrity of the aortic root persists through the full root technique and contributes to the long-term structural stability. On the other hand, re-root replacement can be a hazardous procedure, so that treatment for degenerated roots however may be facilitated by TAVI treatment or implantation of rapid deployment valves in a valve-in-root procedure, described by the authors institution^23^.
